# The potential association of peripheral inflammatory biomarkers in patients with papillary thyroid cancer before radioiodine therapy to clinical outcomes

**DOI:** 10.3389/fendo.2023.1253394

**Published:** 2023-12-15

**Authors:** Jingjia Cao, Xiaoxi He, Xiao Li, Yaru Sun, Wei Zhang, Yuyang Li, Xiaolu Zhu

**Affiliations:** ^1^ Department of Nuclear Medicine, the Second Hospital of Shandong University, Jinan, China; ^2^ Cheeloo College of Medicine, Shandong University, Jinan, China; ^3^ Department of Breast and Thyroid Surgery, Shandong Provincial Hospital Affiliated to Shandong First Medical University, Jinan, Shandong, China; ^4^ Department of Breast and Thyroid Surgery, Shandong Provincial Hospital, Shandong University, Jinan, China

**Keywords:** inflammatory biomarker, lymphocyte, papillary thyroid cancer, radioiodine, therapy

## Abstract

**Purpose:**

Neutrophil-lymphocyte ratio (NLR), markers-lymphocyte-to-monocyte ratio (LMR), and platelet to-lymphocyte ratio (PLR) have potential roles as prognostic biomarkers in various cancers. The study was evaluated to investigate the predictive value of the peripheral inflammatory biomarkers in patients with papillary thyroid carcinoma (PTC) before radioiodine therapy to the response of clinical outcomes.

**Methods:**

We retrospectively analyzed the patients diagnosed with PTC at the Second Hospital of Shandong University between September 2018 and January 2020. Patients were divided into low and high inflammatory biomarker groups based on median values. The area under the receiver operating characteristic curves (ROC) and logistic regression were used to explore the potential risk factors.

**Results:**

A total of 692 patients were enrolled, which included 197 (28.4%) males and 495 (71.6%) females. The median values of NLR, LMR and PLR of these patients were 1.7 (range 0.3–5.7), 7.1 (range 1.1–23.4) and 137.6 (range 27.6–497.5), respectively, and the mean values were 1.95 ± 0.82, 7.4 ± 2.5 and 148.7 ± 54.8, respectively. Compared to the lower PLR group, the higher group was significantly associated with gender, tumor size, N stage and thyroglobulin level (*P*<0.05). At the end of follow-up, 75.5% (523/692), 13.3% (91/692), 4.5% (31/692), and 6.7% (47/692) of patients were evaluated as excellent response (ER), indeterminate response (IDR), structural incomplete response (SIR), and biochemical incomplete response (BIR) respectively. In term of clinical outcomes, the NLR, LMR and PLR showed relatively low discriminative power (*P*≥0.05).

**Conclusion:**

We found that higher PLR values was associated with poor clinicopathological features in PTC. However, the peripheral inflammatory indicators (NLR, LMR and PLR) may be insufficient to predict short-term clinical outcomes of patients with radioiodine therapy.

## Introduction

The number of thyroid cancer continues to increase globally, and most is the papillary thyroid cancer (PTC) (1). Given that the most PTC retains the expression of sodium iodide symporter (NIS), radioiodine (RAI) therapy can eliminate residual and metastatic lesions after thyroid surgery (2). Despite the fact that PTC were generally considered to be with excellent response to RAI therapy, but not all patient. If through some indicators of patients before RAI therapy to predict the response, it would help us to adjust the treatment plan in time and avoid overtreatment and adverse reactions. The prediction of poor clinical outcome in previous studies mainly focused on genetic mutations and thyroglobulin levels. Therefore, the necessity of evaluating biomarkers to enhance the predictive ability is evident.

Notably, a growing body of evidence suggested that cancer-related peripheral inflammation was associated with carcinogenesis and progression in a variety of malignancies, including papillary thyroid cancer (3). Neutrophils, lymphocytes and monocytes in the tumor microenvironment are important biomarkers of tumor related inflammation. In theory, neutrophils promote the growth and invasion of tumor cells by producing some inflammatory factors, and platelets can help tumor cells to evade anti-tumor immunity. Furthermore, lymphocyte infiltration reflects the progression of the tumor and the state of the patient’s immune system (4).

Although available conclusions were inconsistent, many studies had demonstrated that the inflammatory markers-lymphocyte-to-monocyte ratio (LMR), neutrophil-lymphocyte ratio (NLR) and platelet to-lymphocyte ratio (PLR) may have potential role as prognostic biomarkers (5). However, the predictive value of inflammatory biomarkers for response to RAI therapy was still unclear, and related studies were rare. Therefore, we aimed to investigate the predictive value of the peripheral inflammatory biomarkers in patients with PTC before RAI therapy to the response of clinical outcomes.

## Materials and methods

### Study patients

We conducted a cohort study of 945 patients with a pathological diagnosis of PTC who received RAI therapy at the Second Hospital of Shandong University, between September 2018 and January 2020. This single-center, retrospective study protocol received approval from the institutional review board of our institute (KYLL-2022LW078), and involved human subjects in accordance with the Helsinki Declaration. This study has been registered with the Chinese Clinical Trial Registry (ChiCTR2200062911).

**Figure 1:**
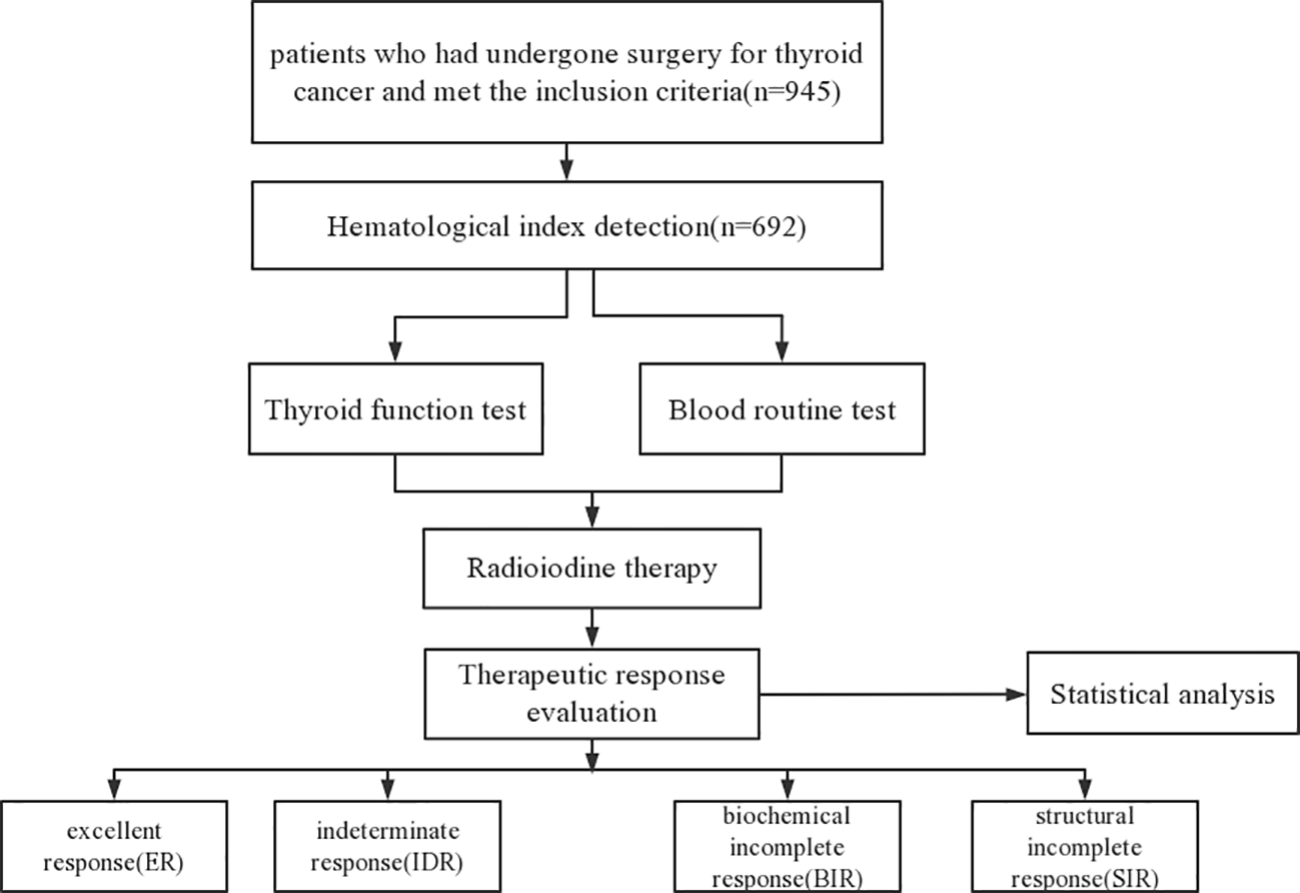
Flow diagram of the patients included in the current study.

The inclusion criteria were as follows: (1) patients with papillary thyroid cancer who underwent thyroidectomy and/or neck lymph node dissection; (2) patients with intermediate- or high-risk of tumor recurrence. The exclusion criteria were as follows: (1) patients with low risk of tumor recurrence; (2) patient with infectious disease; (3) patients with hematologic diseases; (4) patients with immune system disease or other tumors; (5) patients with insufficient follow-up data. Flow diagram of the patients included in the current study is presented in **Figure 1**.

### Inflammatory biomarkers

Laboratory data were measured before the day of RAI therapy. With a fasting more than 8 hours, blood sampling was performed in the morning as biochemical tests between 08:00 and 10:00 am. The inflammatory indices were calculated based on the preoperative complete blood count. NLR is the ratio of neutrophil count to lymphocyte count, PLR is the ratio of platelet count to lymphocyte count, and LMR is the ratio of lymphocyte count to monocyte count. Preoperative blood counts with leukocyte fractions were counted by flow cytometry using an XN-9000 (Sysmex, Japan). The cut-off value of each inflammatory biomarker was determined based on the median value, and patients were then divided into subgroups according to cut-off value.

### Radioactive iodine therapy

All patients were prepared a standard protocol. Patients were on a strict low-iodine diet and discontinued levothyroxine for at least 2-4 weeks before receiving radioiodine therapy. If the patient had an intermediate risk of recurrence, the patient was given 3700MBq. All patients received L-T4 inhibition therapy (2.0-2.5μg/kg per day according to their body mass) 48-72h after radioiodine treatment, and were regularly followed up for 6-12 months. The therapeutic regimen and procedure of radioiodine-131 therapy was carried out as described previously (6).

### Evaluation of treatment response

Based on serological evidence (thyroglobulin and thyroglobulin antibody) and imaging evidence (radioiodine imaging, ultrasound, CT scanning, MRI and PET/CT imaging), the response to radioiodine therapy were classified as excellent response (ER), indeterminate response (IDR), biochemical incomplete response (BIR) and structural incomplete response (SIR). Specific dynamic evaluation criteria were detailed in ATA guidelines (2015). The definition of ER refers to patients with negative imaging, TgAb, and either suppressed Tg < 0.2 ng/ml or stimulated Tg (sTg) < 1 ng/ml.

### Statistical analyses

The results are expressed as mean ± SD for continuous data and percentage (%) for categorical data. Both cohorts were compared using the χ2-test or Fisher’s exact test for categorical variables or the Wilcoxon Mann-Whitney test for continuous variables. Pearson or Spearman rank correlation was used for correlation analysis of data distribution. Receiver operating characteristic (ROC) curve analysis was performed to evaluate the prognostic values of inflammation. Logistic regression was used to analyze and predict ER risk factors at the end of follow-up. *P* < 0.05 was considered to be statistically significant.

## Results

### Baseline characteristics of patients

A total of 692 patients were analyzed in this study after excluding 253 patients for the following reasons: 123 patients with low risk of tumor recurrence, 23 patients had infectious diseases, and 107 patients without sufficient follow-up data. 692 patients included 197 (28.4%) males and 495 (71.6%) females, and the mean age was 43.5 ± 11.7 years (range, 18–72 years). Of these, 131 patients (18.9%) were older than 55 years, whereas 561 patients (81.1%) were less than 55 years of age. 379 (54.7%) patients had multifocal lesions, and advanced T stages (T3+T4) occurred in 46 (6.6%) patients. In addition, 348 (50.3%) patients had lateral cervical lymph nodes metastasis. At baseline, the median values of NLR, LMR and PLR of these patients were 1.7 (range 0.3–5.7), 7.1 (range 1.1–23.4) and 137.6 (range 27.6–497.5), respectively, and the mean values were 1.95 ± 0.82, 7.4 ± 2.5 and 148.7 ± 54.8, respectively. The mean follow-up period was 36 months. The baseline characteristics of patients were listed in **Table 1**.

**Table 1:** Baseline characteristics of 692 patients.

Characteristics	Total (n=692)
Age at diagnosis (years)	43.5 ± 11.7
<55	561, 81.1%
≥55	131, 18.9%
Gender, female (n, %)	495, 71.5%
Multifocality (yes, n, %)	379, 54.7%
Hashimoto’s thyroiditis (yes, n, %)	107, 15.5%
Tumor size (cm)	1.2 ± 1.0
T stage (n, %)	
T1+T2	646, 93.4%
T3+T4	46, 6.6%
N stage (n, %)	
N0	23, 3.4%
N1a	321, 46.3%
N1b	348, 50.3%
Baseline laboratory data	
Neutrophil (%)	58.9 ± 8.9
Lymphocyte (%)	33.4 ± 8.1
Monocyte (%)	4.8 ± 1.2
NLR	1.7(1.3, 2.3)
LMR	7.1(5.6, 8.7)
PLR	137.6(114.3, 171.7)
Thyroglobulin (ng/mL)	2.1(0.4, 6.9)
TSH level (mIU/L)	107.1(78.9, 137.6)
TPOAb level (IU/mL)	0.9(0.4, 2.5)
TGAb level (IU/mL)	0(0, 0.5)

LMR, lymphocyte/monocyte ratio; NLR, neutrophil/lymphocyte ratio; PLR, platelet/lymphocyte ratio; TSH, thyroid-stimulating hormone; TPOAb, thyroid peroxidase antibody; TGAb, thyroglobulin antibody.

### Clinical parameters based on inflammatory biomarkers

The analysis showed a correlation between NLR, PLR and the N stage (r=0.08, *P*=0.03; r=0.09, *P*=0.01). And there was also significant correlation between LMR, PLR and gender (r=0.08, *P*=0.03; r=0.15, *P*=0.01) (**Table 2**). Patients were divided into two groups based on the median level of inflammatory biomarkers respectively. Compared with the different NLR groups, there were no significant differences in age, gender, and other clinical factors. However, the LMR values were significantly associated with gender (*P*=0.03) between two group. Compared to the lower PLR group, the higher group was significantly associated with gender, tumor size, N stage and thyroglobulin level (**Table 3**).

**Table 2:** The relationship of inflammatory biomarkers with other factors.

	LMR		NLR		PLR	
	correlation r	*P* value	correlation r	*P* value	correlation r	*P* value
Age (years)	-0.01	0.63	-0.07	0.06	-0.01	0.81
Gender (n)	0.08	**0.03**	-0.06	0.08	0.15	**0.01**
Multifocality (n)	0.02	0.51	0.00	0.99	0.02	0.52
Hashimoto’s thyroiditis (n)	0.01	0.78	-0.02	0.47	0.03	0.32
Tumor size (cm)	-0.03	0.43	0.02	0.58	-0.03	0.37
T stage (n)	-0.01	0.71	-0.01	0.64	0.01	0.91
N stage (n)	0.00	0.99	0.08	**0.03**	0.09	**0.01**
Thyroglobulin (ng/mL)	-0.04	0.27	0.01	0.71	-0.02	0.51
TSH level (mIU/L)	0.07	0.05	-0.05	0.12	-0.02	0.62

LMR, lymphocyte/monocyte ratio; NLR, neutrophil/lymphocyte ratio; PLR, platelet/lymphocyte ratio; TSH, thyroid-stimulating hormone.

The bold label means statistically significant difference.

**Table 3:** Clinical parameters of patients based on inflammatory biomarkers.

Characteristics	LMR			NLR			PLR		
	<7.1 (n=347)	≥7.1 (n=345)	*P*	<1.7 (n=320)	≥1.7 (n=372)	*P*	<137.6 (n=346)	≥137.6 (n=346)	*P*
Age (years)	43.6 ± 11.3	43.4 ± 12.1	0.81	43.5 ± 12.2	43.4 ± 11.3	0.89	42.9 ± 11.1	44.1 ± 12.3	0.19
Gender (n, %)									
Male	111(32.0%)	86(24.9%)	**0.04**	83(25.9%)	114(30.6%)	0.17	73(21.1%)	124(35.8%)	**0.01**
Female	236(68.0%)	259(75.1%)		237(74.1%)	258(69.4%)		273(78.9%)	222(64.2%)	
Multifocality (n, %)									
No	161(46.4%)	152(44.1%)	0.53	154(48.1%)	159(42.7%)	0.15	151(43.6%)	162(46.8%)	0.41
Yes	186(53.6%)	193(55.9%)		166(51.9%)	213(57.3%)		195(56.4%)	184(53.2%)	
HT (n, %)									
No	294(84.7%)	292(84.6%)	0.43	265(82.8%)	321(86.3%)	0.36	279(80.6%)	307(88.7%)	0.07
Yes	53(15.3%)	53(15.4%)		55(17.2%)	51(13.7%)		67(19.4%)	39(11.3%)	
T stage (n, %)									
T1	286(83.4%)	280(81.2%)	0.63	262(81.9%)	304(81.7%)	0.92	283(81.8%)	283(81.8%)	0.32
T2	36(10.4%)	44(12.8%)		37(11.6%)	43(11.6%)		35(10.1%)	45(13.0%)	
T3	18(5.2%)	17(4.9%)		15(4.7%)	20(5.4%)		21(6.1%)	14(4.0%)	
T4	7(2.0%)	4(1.2%)		6(1.9%)	5(1.3%)		7(2.0%)	4(1.2%)	
Tumor size (cm)									
<1	147(42.4%)	142(41.2%)	0.74	129(40.3%)	160(43.0%)	0.47	160(46.2%)	129(37.3%)	**0.01**
≥1	200(57.6%)	203(58.8%)		191(59.7%)	212(57.0%)		186(53.8%)	217(62.7%)	
N stage (n, %)									
N0	11(3.2%)	12(3.5%)	0.71	13(4.1%)	10(2.7%)	0.08	13(3.8%)	10(2.9%)	0.05
N1a	165(47.8%)	155(44.9%)		160(50.0%)	161(43.3%)		175(50.6%)	146(42.2%)	
N1b	170(49.0%)	178(51.6%)		147(45.9%)	210(54.0%)		158(45.7%)	190(54.9%)	
Thyroglobulin (ng/mL)									
<10	279(80.4%)	276(80.0%)	0.89	248(77.5%)	307(82.5%)	0.09	288(83.2%)	267(77.2%)	**0.04**
≥10	68(19.6%)	69(20.0%)		72(22.5%)	65(17.5%)		58(16.8%)	79(22.8%)	
1-year response (n, %)									
ER	179(51.6%)	194(56.2%)	0.44	175(54.7%)	198(53.2%)	0.91	190(54.9%)	183(52.9%)	0.43
IDR	73(21.0%)	73(21.2%)		69(21.6%)	77(20.7%)		70(20.2%)	76(22.0%)	
SIR	63(18.2%)	48(13.9%)		49(15.3%)	62(16.7%)		60(17.3%)	51(14.7%)	
BIR	32(9.2%)	30(8.7%)		27(8.4%)	35(9.4%)		26(7.5%)	36(10.4%)	
3-year response (n, %)									
ER	257(74.1%)	266(77.1%)	0.74	241(75.3)	282(75.8%)	0.75	257(74.3%)	266(76.9%)	0.62
IDR	48(13.8%)	43(12.5%)		43(13.4%)	48(12.9%)		46(13.3%)	45(13.0%)	
SIR	18(5.2%)	13(3.8%)		12(3.8%)	19(5.1%)		19(5.5%)	12(3.5%)	
BIR	24(6.9%)	23(6.7%)		24(7.5%)	23(6.2%)		24(6.9%)	23(6.6%)	

HT, Hashimoto’s thyroiditis; LMR, lymphocyte/monocyte ratio; NLR, neutrophil/lymphocyte ratio; PLR, platelet/lymphocyte ratio; ER, Excellent response; IDR, indeterminate response; BIR, biochemical incomplete response; SIR, structural incomplete response.

The bold label means statistically significant difference.

### Associations between inflammatory biomarkers and clinical outcomes

**Figure 2:**
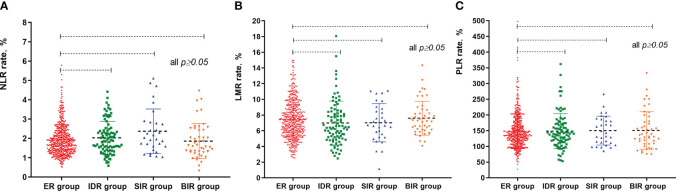
The different median values of NLR, LMR and PLR between these patients according to radioactive iodine therapy outcome. **(A)** scatter plot of NLR values between different groups; **(B)** scatter plot of LMR values between different groups; **(C)** scatter plot of PLR values between different groups.

**Figure 3:**
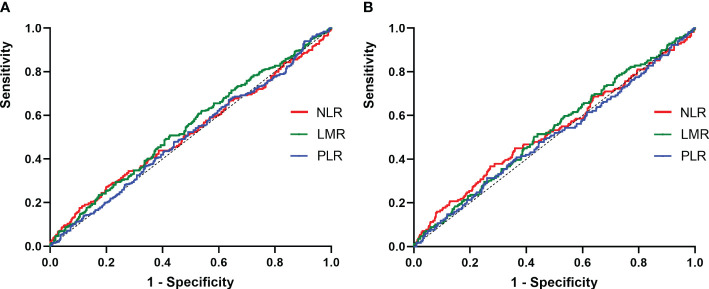
ROC curves for the preoperative NLR, LMR and PLR to predict non-ER. **(A)** 1-years response, the AUC of the NLR for predicting non-ER was 0.51 (95% CI, 0.47-0.56; p=0.39), and the AUC of the LMR for predicting non-ER was 0.54 (95% CI, 0.49-0.58; p=0.05), and the AUC of the PLR for predicting non-ER was 0.51 (95% CI, 0.46-0.55; p=0.75); **(B)** 3-years response, the AUC of the NLR for predicting non-ER was 0.52 (95% CI, 0.47-0.58; p=0.24), and the AUC of the LMR for predicting non-ER was 0.53 (95% CI, 0.48-0.58; p=0.22), and the AUC of the PLR for predicting non-ER was 0.51 (95% CI, 0.43-0.55; p=0.89).

At the end of follow-up, 75.5% (523/692), 13.3% (91/692), 4.5% (31/692), and 6.7% (47/692) of patients were evaluated as ER, IDR, SIR and BIR respectively. Based on each inflammatory biomarker, ROC curve analysis was conducted to predict the clinical outcomes, respectively (**Figures 2**, **3**). In term of clinical outcomes, the NLR, LMR and PLR showed relatively low discriminative power. In univariate logistic regression analysis, the roles of gender (*OR*=0.43, *P*=0.01), Hashimoto’s thyroiditis (*OR*=2.52, *P*=0.03), tumor size (*OR*=1.37, *P*=0.01), and thyroglobulin level (*OR*=1.16, *P*=0.01) were significant in distinguishing between the ER and non-ER (IDR, BIR and SIR) at the end of follow-up. In further multivariate logistic regression analysis, gender (*OR*=0.45, *P*=0.01) and thyroglobulin level (*OR*=1.15, *P*=0.01) were confirmed to be independent factors (**Table 4**).

**Table 4:** Univariate and multivariate analyses of the risk factors for clinical outcomes.

	Univariate			Multivariate		
	*OR*	*95%CI*	*P*	*OR*	*95%CI*	*P*
Gender						
Male	Ref.					
Female	0.43	0.31-0.61	**0.01**	0.45	0.31-0.65	**0.01**
Age(years)	0.98	0.97-1.00	0.07			
<55	Ref.					
≥55	0.91	0.62-1.33	0.64			
Multifocality	0.93	0.69-1.26	0.67			
Hashimoto’s thyroiditis	2.52	1.30-3.62	**0.03**	1.23	0.77-1.97	0.37
Tumor size	1.37	1.16-1.62	**0.01**	1.15	0.84-1.58	0.35
T stage						
T1	Ref.			Ref.		
T2	1.41	0.88-2.26	0.14	0.84	0.37-1.88	0.67
T3	2.17	1.07-4.39	0.03	1.11	0.41-3.07	0.83
T4	2.24	0.65-7.75	0.21	2.11	0.51-8.72	0.31
N stage						
N0	Ref.			Ref.		
N1a	0.96	0.41-2.30	0.72	0.94	0.35-2.51	0.91
N1b	1.81	0.46-2.78	0.78	1.27	0.48-3.38	0.62
Thyroglobulin level						
<10	Ref.			Ref.		
≥10	1.16	1.12-1.20	**0.01**	1.15	1.11-1.19	**0.01**
Inflammation index						
LMR	0.94	0.89-1.01	0.07			
NLR	1.17	0.98-1.41	0.07			
PLR	1.00	0.99-1.00	0.72			

HT, Hashimoto’s thyroiditis; LMR, lymphocyte/monocyte ratio; NLR, neutrophil/lymphocyte ratio; PLR, platelet/lymphocyte ratio.

The bold label means statistically significant difference.

## Discussion

Tumor related inflammation played an important role in the development and progression of cancer (7). Recently, more and more studies have shown that inflammatory indicators (such as NLR, LMR, PLR, etc.) have important predictive value in the prognosis of many malignant tumors (8). The neutrophil to lymphocyte ratio was one of the early inflammatory indices used to predict the prognosis of thyroid cancer (9). Although the role of NLR has not been fully proved in PTC with regard to clinical outcome, several reports indicated that a higher NLR was associated with worse outcome (10). To the best of our knowledge, there has been no studies investigating the association between LMR, PLR and RAI therapy in patients with papillary thyroid cancer.

In our study we retrospectively analyzed 692 patients. Based on the data obtained, the median values of NLR, LMR and PLR of these patients were 1.7 (range 0.3–5.7), 7.1 (range 1.1–23.4) and 137.6 (range 27.6–497.5), respectively, and the mean values were 1.95 ± 0.82, 7.4 ± 2.5 and 148.7 ± 54.8, respectively. Compared with other research, the LMR values were high, and the NLR and PLR values were more similar (11). In our study, the average age was 43.5 ± 11.7 years, and most were female (71.5%), which might relate to the high incidence of thyroid cancer in women. Most of the patients did not have Hashimoto’s thyroiditis. In addition, most of the patients’ stages were T1/2, and this was similar to the basic clinicopathological features of patients enrolled in other institutes (12, 13).

Overall, a higher NLR values indicates more aggressive behavior and more advanced disease stage of tumor, manifested as larger size, multifocality, and lymph node metastases. Unfortunately, we did not confirm this association in the present study. Moreover, it is worth mentioning that the association between carcinoma and chronic inflammation had been well-studied. However, our series could not establish connection between NLR and Hashimoto thyroiditis (1.91 ± 0.83 with *vs* 1.95 ± 0.82 without thyroiditis, *P*=0.59). Based on the median value, a diagnostic cutoff of 1.7 is proposed to distinguish low and high NLR group. A retrospective study suggested that the recurrence rate was lower in the high NLR group (10.8% *vs* 15.9%), when the threshold was 1.6. While Kim et al. showed worse clinical outcomes in stage III-IV patients with an NLR cutoff of 1.5, Lang et al. and Cho et al. found that NLR determined neither disease-free survival nor cancer-specific death rates (14). The emergence of different conclusions may be due to certain heterogeneity in sample sources, sample numbers, pathological subtypes, and other aspects among different studies. In addition, a comprehensive meta-analysis was displayed that a higher NLR (median cutoff 4.0) was related to an adverse outcomes in many solid tumors (15). However, only 18 patients (2.6%) presented with ratios greater than 4.0. And 77 patients (11.1%) presented with ratios greater than 3.0. Among these patients, 33 patients (42.8%) reached ER status at the initial treatment, and 49 patients (63.6%) reached ER status at the follow-up (third year).

The LMR may present tumor-related lymphocyte and tumor-associated macrophages, and the later promote tumor progression and inhibit tumor elimination. It is reasonable to assume the connection between lower LMR and poor clinical outcome (16). In our cohort, the LMR value was associated with gender (female). In addition, advanced age (≥45 years) has been reported previously correlated with higher LMR values. In our series, advanced age (≥55 years) had a slightly higher LMR values (7.38 ± 2.56) compared to <55 years (7.25 ± 2.42), and the difference was not significant. Yokota et al. found that the higher LMR values (≥5.0), as the optimal cutoff point, had a higher recurrence free survival rate by plotting the ROC curve of LMR (17). Kim et al. also found that the high LMR group with an optimal cutoff point of 7.05 had higher recurrence-free survival (18). However, an elevated LMR values were not associated with an increased ER rates in our study.

Several studies had indicated that higher PLR values were associated with poor outcomes (19). In our study, the increased PLR values were associated with gender, tumor size (≥1cm) and lymph node metastasis. This observation was consistent with the results of JIANG K et al. indicating that the high levels of PLR in agreement with poor feature (20). But it was not related to other factors, such as multifocal and Hashimoto’s thyroiditis (all *P*≥0.05). Moreover, we also found that higher PLR values were associated with higher levels of thyroglobulin. Our results are consistent with those reported previously, further suggesting the strong predictive ability of the LMR. On the contrary, Ceylan et al. found that there was no association between the clinicopathological features and the PLR values (21). Because the PLR AUC was 0.51, and we did not find a cut-off value for PLR to predict clinical outcomes in our study. Lee et al. retrospectively analyzed 914 Korean PTC patients and indicated that the disease-free survival was longer in the lower PLR group when using a threshold of 164.24 (22). However, a retrospective analysis of 1873 Chinese patients with PTC found that PLR values did not have a significant predictive effect on recurrence (23). The specific mechanism is not yet clear, and it may be the release of various inflammatory mediators that induce an increase in platelets.

There was no significant difference in NLR, LMR and PLR values between the ER and the non-ER groups in the present study. The results of our study were negative, and the inflammatory indicators before the first radioactive iodine therapy had no predictive value for its curative effect. In brief, this phenomenon might be attributed to the fact of thyroid cancer characteristic. As an indolent cancer, thyroid cancer may be less sensitive to changes in tumor microenvironment and inflammatory indicators in peripheral blood than other malignant tumors. Therefore, the predictive value of inflammatory indicators for the prognosis and therapeutic effect of thyroid cancer may not be as high as that of other malignant tumors. Moreover, due to the good prognosis of thyroid cancer, the inflammatory index values are expected to be low and within a narrow range, which can also make it not significantly different between the satisfactory and unsatisfactory treatment groups. The other issue probably resides in the fact that more than 80% of the enrolled patients were T1a. It has been already demonstrated that NLR and PLR are higher in patients with worst pathological features and larger tumors. Thus, T1a patients are probably not the right population in which these biomarkers can predict the outcome after RAI therapy.

There are several limitations to the present study. Firstly, due to the retrospective nature of the study, there may be a possibility of selection bias. Secondly, we are unable to evaluate the changes in each inflammatory biomarker during RAI treatment. Thirdly, we used the median of each inflammatory biomarker as the cutoff value, which may be difficult to apply to other studies. Finally, our study protocol was designed to address the issue of NLR in conjunction with patient ER rates and to propose specific cutoff values. Given the generally excellent 5-year and 10-year disease-free and overall survival of PTC patients, a long-term follow-up would be required to draw safe, meaningful conclusions.

In conclusion, we found that higher PLR values was associated with poor clinicopathological features in PTC. However, the peripheral inflammatory indicators (NLR, LMR and PLR) may be insufficient to predict short-term clinical outcomes of patients with radioiodine therapy.

## Data availability statement

The raw data supporting the conclusions of this article will be made available by the authors, without undue reservation.

## Ethics statement

The studies involving humans were approved by the Second Hospital of Shandong University (KYLL-2022LW078). The studies were conducted in accordance with the local legislation and institutional requirements. The participants provided their written informed consent to participate in this study.

## Author contributions

JC: Conceptualization, Writing – original draft. XH: Data curation, Writing – review & editing. XL: Data curation, Methodology, Writing – review & editing. YS: Writing – review & editing. WZ: Data curation, Writing – review & editing. YL: Conceptualization, Resources, Writing – review & editing. XZ: Data curation, Writing – review & editing.
